# *Staphylococcus aureus* Endocarditis Immunothrombosis

**DOI:** 10.3390/metabo15050328

**Published:** 2025-05-15

**Authors:** Francesco Nappi

**Affiliations:** Department of Cardiac Surgery, Centre Cardiologique du Nord, 93200 Saint-Denis, France; francesconappi2@gmail.com; Tel.: +33-149334104; Fax: +33-149334119

**Keywords:** infective endocarditis, *Staphylococcus aureus* infection, platelet activation, thromboimmunity, *Staphylococcus aureus*, immunoresponse

## Abstract

**Background:** Infective endocarditis continues to represent a challenge for healthcare systems, requiring careful management and resources. Recent studies have indicated a shift in the predominant pathogens of concern, with *Streptococcus* sp. a being superseded by *Staphylococcus* sp. and *Enterococcus* sp. as the leading causes of concern. This shift is of concern as it is associated with *Staphylococcus Aureus* which has a high virulence rate and a tendency to form a biofilm, meaning that non-surgical therapy may not be effective. It is imperative to deliberate on the likelihood of platelet blood clot formation, which may be accompanied by bacterial infestation and the development of a biofilm. **Methods:** MEDLINE, Embase, and Pubmed were searched using terms relating to ‘endocarditis’ and ‘*Staphilococcus aureus*’, along with ‘epidemiology’, ‘pathogenesis’, ‘coagulation’, ‘platelet’, ‘aggregation’, and ‘immunity’. The search focused on publications from the past 15 years, but excluded older, highly regarded articles. We also searched the reference lists of relevant articles. Recommended review articles are cited for more details. **Results:** An endocarditis lesion is believed to be a blood clot infected with bacteria that adheres to the heart valves. Infective endocarditis is a good example of immunothrombosis, where the coagulation system, innate immunity and the function of coagulation in isolating and eliminating pathogens interact. However, in the context of infective endocarditis, immunothrombosis unintentionally establishes an environment conducive to bacterial proliferation. The process of immunothrombosis impedes the immune system, enabling bacterial proliferation. The coagulation system plays a pivotal role in the progression of this condition. **Conclusion:** The coagulation system is key to how bacteria attach to the heart valves, how vegetations develop, and how complications like embolisation and valve dysfunction occur. *Staphylococcus aureus*, the main cause of infective endocarditis, can change blood clotting, growing well in the fibrin-rich environment of vegetation. The coagulation system is a good target for treating infective endocarditis because of its central role in the disease. But we must be careful, as using blood-thinning medicines in patients with endocarditis can often lead to an increased risk of bleeding.

## 1. Introduction

Infective endocarditis (IE) is now more challenging than ever for healthcare providers. The reasons for this are numerous. Among the demographic groups most impacted is the elderly population, who often exhibit multiple comorbid conditions and have reduced physiologic reserves in comparison to previous cohorts [1,2,3,4,5]. In the absence of intervention, IE result in a mortality rate approaching 100%, and even with optimal treatment, the mortality rate remains at 33% [6].

In recent years, virulent strains of staphylococcus have gradually overtaken streptococcal strains in the epidemiological significance of IE. This shift is particularly notable in many high-income countries, where penicillin sensitivity, historically a characteristic of streptococcal infections, has become less prevalent. However, it is important to point out that unlike other infectious and cardiovascular disorders, its high mortality rate has not changed over the course of the last few decades. Despite its relatively infrequent incidence, with an annual prevalence ranging from 3 to 10 per 100,000 individuals [1,2,7,8,9], IE has been identified as a significant cause of mortality, responsible for more than 100,000 deaths annually worldwide [10]. Consequently, IE has emerged as a major health concern, particularly in the context of healthcare settings, where the population at risk for IE has significantly expanded. The shift towards virulent strains of staphylococcus is accompanied by a heightened awareness of the risk of staphylococcal bacteremia, underscoring the need for ongoing research and vigilant clinical monitoring. This phenomenon currently poses the most significant global challenge, as it is the primary catalyst for the development of IE [10,11]. The rise in antibiotic resistance is a grave concern in modern healthcare, as it poses a significant threat to public health [12,13,14].

Consequently, there is an urgent need for novel strategies for the treatment and prevention of this disabling condition. The pathogenesis of the condition under investigation is so complex and so unique, that it remains to this day not entirely clear how and why certain strains of bacteria, and not their counterparts, flourish in an environment so inhospitable as the cardiac valves. This review aims to elucidate the central yet enigmatic function of the coagulation system in the diverse stages of endocarditis.

## 2. Methods

A comprehensive search was conducted on the following databases: MEDLINE (https://www.nlm.nih.gov/medline/medline_overview.html; 8 May 2025), Embase (https://www.embase.com/landing?status=yellow; 8 May 2025), and PubMed (https://pubmed.ncbi.nlm.nih.gov/; 8 May 2025). The search terms “endocarditis” or “infective endocarditis” were entered together with the term “*Staphylococcus aureus*”, along with the following additional keywords: “epidemiology”, “pathogenesis”, “coagulation”, “platelet”, “aggregation”, and “immunity”. A selection of publications from the past 15 years was made, but older publications that have been widely referenced and held in high esteem were not excluded. A further step in the research process involved the examination of the reference lists of articles that had been identified by the search strategy. The selection of articles for further analysis was based on a judgement of relevance. In order to furnish readers with further details and background references, recommended review articles are cited.

## 3. Results

### 3.1. The Focus of the Infection

**Figure 1:**
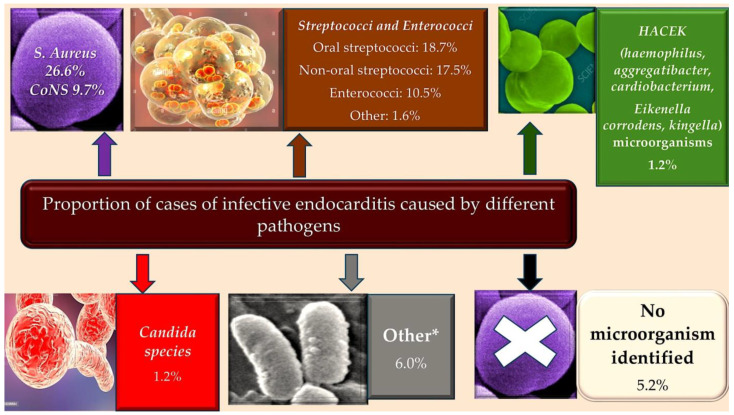
The prevalence of IE has been observed to occur most frequently among the elderly with a history of CIEDs, as well as among younger individuals with a history of IDVU. Conversely, a lower incidence has been documented in patients with central venous catheters, HIV, CHD, and those experiencing immunosuppression. * Low numbers of *Coxiella burnetii*, *Bartonella quintana*, *Pseudomonas aeruginosa*, *Tropheryma whipplei*, *Enterobacteriaceae*, *Acinetobacter ursingii*, *Listeria monocytogenes*, *Propionibacterium acnes*, *Lactobacillus* spp., *Corynebacterium* spp., *Francisella tularensis*, *Erysipelothrix rhusiopathiae*, *Gordonia bronchialis*, *Bacillus* spp., *Catabacter hongkongensi*, *Moraxella catarrhalis*, *Campylobacter foetus*, *Neisseria elongata* and *Veillonella* spp. Abbreviations: CIED, cardiac implantable electronic devices; CHD, congenital heart disease; CoNS, coagulase negative; HIV, immunodeficiency virus; IDVU, intravenous drug user; IE, infective endocarditis. * This information has been derived from the work of Selton-Suty C et al. Clin Infect Dis 2012; 54: 1230–39. Ref. [15]. Figure from Nappi et al. Ref. [15].

Infective endocarditis, a bacterial infection of the heart, is primarily caused by Gram-positive cocci from the *Staphylococcus*, *Streptococcus*, and *Enterococcus* species. This bacterium is responsible for approximately 80% of cases of IE Figure 1 [15].

In high-income settings, *Staphylococcus aureus* has emerged as the predominant causal pathogen, accounting for up to 30% of infection cases [1,2].

The impact of IE determined by Staphylococcal foci is extensive, affecting numerous individuals across diverse demographics. These include individuals who are traditionally considered high-risk, such as patients undergoing haemodialysis treatment and intravenous drug users. Additionally, the infection can be transmitted to individuals with native, prosthetic valves, and those with cardiac implantable electronic devices (CIEDs) [16,17,18,19,20,21,22]. In addition, *Staphylococcus* cocci exhibits a profound proclivity for acquiring antibiotic resistance, leading to the emergence of meticillin-resistant strains, which have emerged as a grave global concern [23,24,25]. The taxonomic classification of coagulase-negative staphylococci (CoNS) encompasses a diverse array of species, including *Staphylococcus epidermidis*, *Staphylococcus lugdunensis*, and *Staphylococcus capitis*. These organisms are notable for their pervasive presence as commensals on the skin. CoNS possess a range of distinctive characteristics that set them apart from other staphylococcal species. It has been demonstrated that these organisms are especially proficient at colonising indwelling catheters and cardiac implantable electronic devices. They are the most prevalent pathogens identified in cases of primary PVL [26,27,28,29,30].

*Enterococcus faecalis* is a commensal bacterium that naturally colonises the human gastrointestinal and biliary tracts. However, it is also a causative agent of infections that can develop in surgical sites, the urinary tract and the bloodstream [31,32,33,34,35,36]. Similarly, Group D streptococci (e.g., *Streptococcus gallolyticus*, *Streptococcus bovis*), which have been reclassified as *Enterococci* spp., are associated with the development of IE in conjunction with gastrointestinal and urogenital tract pathologies, which utilise the portal venous system as a portal of entry. Recent research in molecular biology and clinical therapeutics has highlighted the significance of the pili in enterococcal infective endocarditis, emphasising its role in bacterial aggressiveness due to biogenesis, host immune response, and resistance to antimicrobial therapy. Research has indicated that F pili fulfil at least three functions in bacterial mating: firstly, by initiating contacts between mating pairs; secondly, by facilitating the transfer of genetic material; and finally, by drawing the mating cells into close proximity, thereby increasing the fertility of the bacterial union [34,35,37,38,39,40,41,42,43,44,45,46]. The latter process may provide a plausible rationale for the predominance of *E. faecalis* as a causative agent of bacterial infective endocarditis, a condition associated with a high mortality rate due to severe complications, including the exacerbation of congestive heart failure, glomerulonephritis, and septic embolism [4,5,6,15,34,35,36].

As demonstrated in the extant literature, the oral viridans group remains the most common causative streptococcal germ [47,48,49,50]. The nomenclature of this group derives from the Latin term ‘viridis’, which translates to ‘green’, a colour that is characteristic of the discolouration of the blood agar medium. Gram-positive cocci of the genus *Streptococcus* include the following species: *Streptococcus mutans*, *Streptococcus salivarius*, *Streptococcus anginosus*, *Streptococcus mitis*, and *Streptococcus sanguinis*. These organisms are classified as commensals of the oral, gastrointestinal, and urogenital tracts.

Gram-negative bacteria, including *Escherichia coli*, account for a considerable proportion of bloodstream infections; however, the incidence of endocarditis caused by these bacteria is low.

### 3.2. The Epidemiology of Infective Endocarditis and the Targeting of Vulnerable Populations to Staphylococcal Infection

The principal concern pertaining to IE pertains to the incongruity that persists among the tendencies towards the prior identification and surgical management of patients with respect to one-year mortality rates, a phenomenon that has remained unimproved for a period spanning over two decennia. This finding suggests that the issue of IE remains a significant issue even though its manifestation has evolved from the pre-antibiotic era to the early phases of targeted antibiotic treatment and finally to the current patient demographic, who have all encountered a variety of microbiome characteristics [51,52]. Historically, IE has been observed in young or middle-aged adults with established rheumatic heart disease or congenital heart disease (CHD). These patient demographics are characterised by a range of risk factors, including but not limited to, prosthetic valve replacement, haemodialysis, venous catheters, immunosuppression, and intravenous (IV) drug abuser [53,54,55,56,57,58,59,60,61]. In the contemporary era, patient profiles are characterised by an augmentation of age, frailty, and comorbidities, which are observed with increasing frequency. Concurrently, *Staphylococcus aureus* has emerged as the predominant causative pathogen, superseding oral streptococci [1,2,3,4,5].

Recent studies have indicated an evolution in the IE trend during the 21st century, resulting in an increased demand for acquired healthcare services, with this phenomenon being observed in over 25% of cases [23,62]. Advancements in the field of cardiology have precipitated substantial changes in patient demographics and the manifestation of diseases. Infective endocarditis exerts a significant impact on CIED [16,63]. The utilisation of percutaneous catheter procedures in the treatment of structural heart disease has the potential to result in elevated rates of infective endocarditis when compared to those observed following the implantation of prosthetic valves, a procedure that employs the conventional surgical approach [63,64,65,66].

The evolution of the epidemiology of IE in high-income countries is associated with significant advancements in the medical and surgical disciplines [4,5,54,55,56,57,58,59,60,61,62]. Consequently, contemporary cases of IE have increased by 25–30% in healthcare facilities. This increase can be attributed to the expansion of medical care provided during hospitalisation or nosocomial admission, or the possibility of contracting infection while receiving outpatient care [1,2,3,4,5,6,23,24,25]. In this particular context, there has been a marked increase in the utilisation of long-term intravenous lines and invasive procedures. These procedures act as conduits for pathogens, leading to elevated rates of staphylococcal bacteremia. Consequently, this is a major precursor to infective endocarditis [67,68,69,70,71]. Recent progressions in cardiological sciences have permitted wider utilisation of prosthetic heart valves and cardiac devices, including permanent pacemakers. Due to their ubiquity, pacemakers are susceptible to infection, owing to their role as a focal point for pathogens. As a result, there has been an increase in recommendations for the implementation of sophisticated devices, including cardiac resynchronisation therapy and implantable cardioverter-defibrillators. This has consequently led to a surge in infection rates associated with cardiac device implantations [17,18,19,71,72]

**Figure 2:**
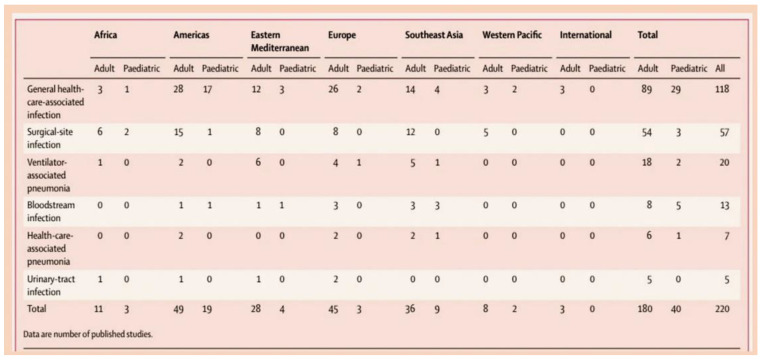
The illustration depicts the prevalence of healthcare-associated infections in developing countries according to the World Health Organisation (WHO) region, patient population, and infection type (1995–2008). Ref. [10].

In such cases, there is a significant need to establish a comprehensive understanding of the epidemiology, pathophysiology, and pathological anatomy of IE, as this is integral to the development of effective strategies for combating it. The investigation will seek to elucidate the factors that have impeded the substantial impact of diagnostics and therapeutic advancements on the course of the illness. As illustrated in Figure 1 and Figure 2, the incidence of IE is documented.

### 3.3. Infective Endocarditis: Could an Immunothrombosis Be the Underlying Cause?

**Figure 3:**
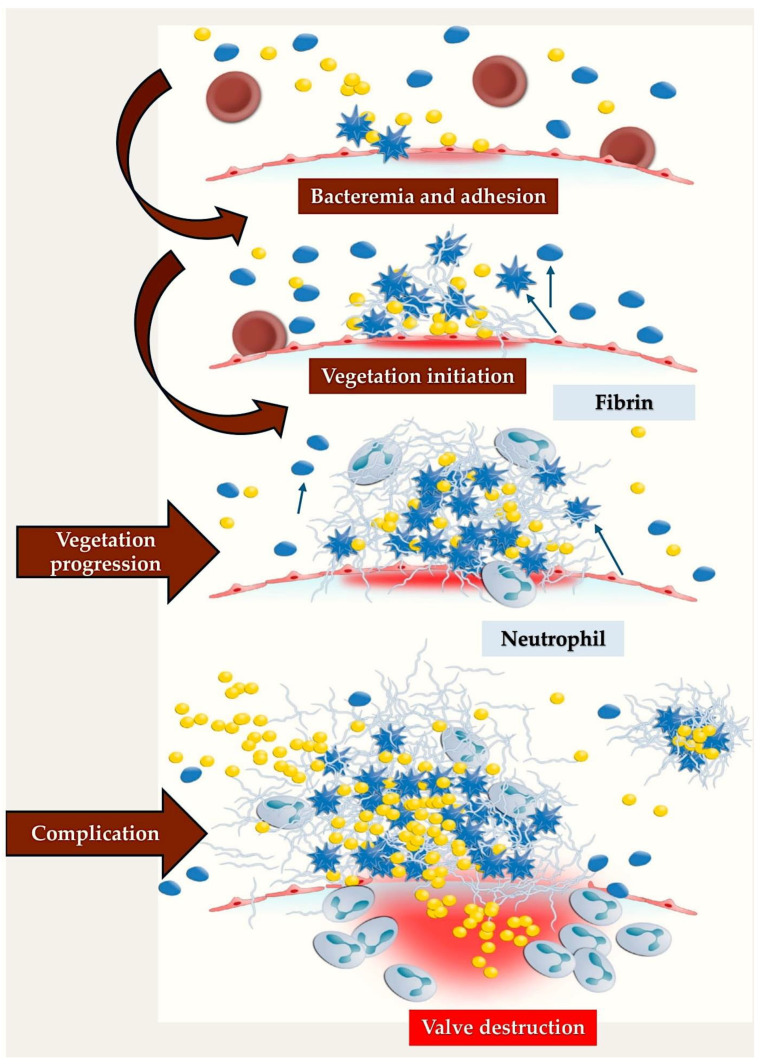
As illustrated by the figure, the progression of endocarditis occurs through distinct phases. Initially, the adhesion of bacterially contaminated blood to the cardiac valve endothelium, either through direct or indirect means, is facilitated by platelets. This initial stage is followed by the migration of platelets and fibrin into the affected area, leading to the formation of a vegetative mass. Concurrently, the immune system, despite the presence of substantial leukocyte concentrations, remains ineffective in impeding the progression of the infection. Consequently, this process can lead to various adverse outcomes, including cardiac valve destruction, embolisation, and uncontrollable sepsis. The blue star-shaped elements represent activated platelets (blue arrows), whereas the blue circle-shaped elements denote inactivated platelets (blue arrows). The yellow elements are representative of bacteria, and the red circle-shaped elements are blood cells.

Cardiac endothelium is not typically exposed to frequent bacteraemia in the instance that no cardiac pathology is present; this is due to the fact that the most common activities which lead to bacteraemia, such as mastication and tooth brushing, are not frequently engaged in [73]. It is a widely accepted notion within the scientific community that bacterial adherence plays a critical role in the pathophysiological process of infective endocarditis. Prevalent microbial agents accountable for the onset of IE tend to proliferate on the valves, accompanied by pre-existing sterile vegetations or valves exhibiting limited endothelial impairment [74]. The inflammatory response initiated in the endothelium is regulated by the synthesis of cytokines, integrins, and tissue factors, which subsequently attract monocytes and platelets, resulting in the synthesis of fibronectin due to the effect of chemokines. These structures facilitate bacterial invasion, which consequently activates the inflammatory cascade. However, the incorporation of bacteria within the host’s defences provides a protective mechanism (Figure 3) [75,76].

IE serves as a prime paradigm illustrating the robust association between the hemostatic mechanism and the innate immune response. This intricate interplay is often designated as immunothrombosis [77]. The formation of an endothelial lesion is known to promote bacterial adhesion, a process that occurs in two stages. Initially, the release of inflammatory cytokines associated with tissue factors leads to the expression of fibronectin, which subsequently facilitates the aggregation of platelets and fibrin, resulting in the formation of a thrombus [74,75,76]. The activation of endothelial cells and leukocytes results in the secretion of tissue factor, thereby triggering the extrinsic coagulation pathway. Concurrently, the intrinsic cascade is initiated through the activation of factor XII by DNA or RNA from necrotic cells or by constituents of bacterial cell walls. Moreover, the intrinsic inflammatory cascade instigates the activation of the pro-inflammatory bradykinin-producing kallikrein system, thereby establishing a linkage between coagulation and the complement system [78,79].

The process of activation of the coagulation cascade ultimately results in the proteolytic cleavage of prothrombin into thrombin. This process is not only vital for maintaining hemostasis but also serves to link coagulation with inflammatory responses. Thrombin has been demonstrated to fulfil multiple functions within the body. In addition to activating platelets and generating fibrin, it also serves to regulate processes of inflammation through the thrombomodulin-protein C pathway and via thrombin receptors located on platelets, endothelial cells, and leukocytes [77]. Thrombin has also been demonstrated to impede the propagation of bacterial infection by occluding the affected tissue with a fibrin layer. Furthermore, peptides that are released during the process of proteolysis have been shown to possess significant antibacterial properties [80]. Simultaneously, platelets, endowed with pattern recognition receptors and immunoglobulin receptors, have gained increasing recognition for their function in innate immunity [81]. These cells are responsible for the elimination of bacteria through the release of platelet microbicidal proteins, which are derived from the alpha-granules. The process of coagulation is further promoted, and the formation of the neutrophil extracellular trap (NET) is stimulated, while platelets, in conjunction with leukocytes, orchestrate the immune response by secreting various cytokines [82,83,84].

Immunothrombosis is a complex system that incorporates numerous regulatory mechanisms to ensure an appropriate response to foreign stimuli. However, in the context of severe infections, this regulatory balance is frequently compromised, leading to the occurrence of complications such as bleeding or thrombosis. Infective endocarditis is a notable example of such a regulatory disturbance. In the aftermath of bacteremia, coagulation is initiated in an effort to neutralise the invading bacteria. However, instead of containing the infection, immunothrombosis engenders an optimal milieu for bacterial proliferation and survival.

### 3.4. Staphylococcus aureus’ Protective Barriers and Host Defence Responses: New Insights from Field Studies and the Role of Coagulases

A substantial body of research utilising diverse animal experiments has revealed that two coagulases, von Willebrand factor binding protein (vWbp) and Coagulase (Coa), play a substantial role in the virulence of the organism.

These proteic substances have been shown to form a complex configuration that *S. aureus* utilises to produce a shield-like structure composed predominantly of fibrinogen/fibrin. The formation of the shield is pivotal to the microorganism’s ability to evade the host’s defensive mechanisms, namely the phagocytic cells that are responsible for this process. A crucial aspect of coagulase function involves the non-proteolytic activation of the zymogen prothrombin, leading to the conversion of fibrinogen into fibrin. This process is instrumental in the formation of a protective shield comprising fibrinogen and fibrin, which contributes to the integrity and stability of the host–microorganism interaction. Coagulases have been shown to exhibit an additional property: namely, their direct interaction with fibrinogen, which significantly contributes to the progression of infection. The binding of vWbp and Coa to fibrinogen involves distinct interactions between the two proteins and the molecule, in spite of the proteins’ similar structure. The adhesion of Coa to soluble fibrinogen exhibited an increased binding affinity in comparison with fibrinogen coated on a plastic surface. Conversely, the vWbp demonstrated no discernible preference between the two structures of fibrinogen [85,86,87,88,89].

Recently, investigative work has yielded significant revelations pertaining to the intricate interactions that occur between fibrinogen and *S. aureus* coagulase. Investigators have posited that vWbp and Coa are each directed towards distinct regions of fibrinogen. Consequently, the presence of these two molecules does not result in the competition for fibrinogen binding sites. It has been established that both Coa and vWbp possess N- and C-terminal regions that are instrumental in mediating their fibrinogen binding capabilities [88,89,90]. The vWbp coagulase has been observed to exhibit an enhanced propensity for binding fibrinogen within the vWbp-N region, a phenomenon that stands in contrast to the Coa, where the highest degree of affinity for the fibrinogen binding site is exhibited within the C-terminal region. Intriguingly, it has previously been reported that the peptides that constitute the previously identified Fibrinogen Coa/Efb1 binding motif are incapable of inhibiting the vWbp-C component from binding to fibrinogen. This finding thus suggests the absence of a functional homologue for this motif in vWbp-C. It is evident that, on further analysis, the N-terminal prothrombin-binding domains of both coagulases appear to recognise the fibrinogen β-chain, yet it would seem that they interact with different sequence motifs in the host protein. The interplay between the two coagulases appeared to be characterised by the expression of distinct sequence motifs within the host protein. The collective analysis of the data yielded a comprehensive perspective on the intricate interplay among Fg and the *S. aureus* coagulases [89,90,91].

The emergence of multidrug-resistant *Staphylococcus aureus* strains has led to significant public health challenges, with these bacteria responsible for the development of life-threatening diseases. The treatment of *S. aureus* infections is hindered by the complexity of the infection process and the absence of an effective vaccine. *S. aureus* possesses the capacity to coat itself with a protective layer composed of fibrinogen and fibrin, thus facilitating its survival and proliferation in adverse conditions. It can be demonstrated that this coating exerts two substantial influences: Firstly, it enables the pathogen to survive in the blood, rendering it invisible to the host’s immune system; secondly, it facilitates the potential for spreading and causing invasive diseases. The modification of this process represents a promising objective for new anti-staphylococcal treatment strategies. However, the mechanisms that characterise it are not yet fully elucidated. *Staphylococcus aureus* expresses a number of proteins that bind to fibrinogen. One study [89] posits the hypothesis that a certain degree of redundancy in the action exerted by some of these proteins with vWbp can serve to limit its function, and that, in the case of proteins which express similar functions, there has often been suggestion of a sharing between them in the structural or functional motif. In their seminal study, Thomas and colleagues [89] made a significant contribution to our understanding of the molecular mechanisms underlying blood platelet aggregation by demonstrating the existence of a protein homologous to the C-terminus of the von Willebrand factor binding protein (vWbp). This discovery provides a crucial insight into the mechanisms of fibrinogen binding and shield assembly. The investigation revealed a common fibrinogen-binding motif shared between vhp and vWbp, thus providing a framework for further exploration of these vital processes [89].

In a recent study, Schwartz et al. undertook a meticulous analysis of the pathomechanisms implicated in the induction of IE. This investigation involved the analysis of 34 isolates of *Staphylococcus aureus*, obtained from subjects afflicted with *S. aureus* endocarditis and from healthy individuals, utilising both in vitro and in vivo models [92]. The study was conducted to assess the in vitro virulence potential of *S. aureus* isolates. The isolates were tested for their capacity to induce tissue destruction (cytotoxicity) and to interact with platelets, which are a vital component of the innate immune response. To establish a correlation between the ability of *S. aureus* to cause vegetations on aortic valves in vivo, the expression profiles of virulence factors and the cellular responses were examined and evaluated using an animal model. This method involved in vivo magnetic resonance imaging at 9.4 T, along with histological evaluation and gene expression analysis, to assess the presence of IE involving valves. *S. aureus* isolates were tested in vitro for their cytotoxicity, invasion potential and interaction with platelets. All in vivo-isolated and -tested *S. aureus* strains demonstrated the capacity to induce IE and the inflammatory response associated with aortic valve injury, but investigators were unable to differentiate and classify IE and inflammation based on the measurement of in vitro virulence profiles and toxicity [92]. Schwartz et al. found no link between in vitro test findings and IE severity. But they discovered significant variations in *Staphylococcus* strains, relating to the extracellular matrix and inflammation. The investigators’ proposal is as follows: Bacteria have different pathogenic capacities, so a comprehensive approach to host–pathogen interactions is necessary to evaluate this. This approach can also study immune pathways to highlight differences in the host/pathogen interaction [92,93,94].

In consideration of the aetiology of *Staphylococcus aureus*-induced infective endocarditis, Schwarz et al. advanced the field by facilitating enhanced comprehension of the interaction between virulence factors and immune responses in *S. aureus*-borne infective endocarditis [92], thereby offering novel prospects for the advancement of therapeutic strategies and the development of specific diagnostic imaging markers [93,94].

### 3.5. Pathogen–Host Interplay in Establishing the Inflammatory Processes

The significance of the impact of *Staphylococcus aureus* on pathogenicity is indisputable. This impact is facilitated by adhesion proteins such as the fibronectin-binding protein and staphylococcal aggregation factors A and B. These proteins act as bacterial mediators of adhesion and are crucial determinants of pathogenicity [95,96,97,98]. A pertinent example is the experimental investigation of *Staphylococcus aureus* adhesins in *Lactococcus lactis*, which demonstrated the pivotal function of ClfA and FnBPA in valve colonisation, as evidenced by the findings from an animal study with experimental heart infection [98,99].

A study was conducted to evaluate the role of progression of infective endocarditis in animals [95]. These animals were observed over a three-day period. It was noted by the investigators that ClfA-positive lactococci successfully colonised damaged valves. However, the observation of spontaneous clearance of infection was made over a 48-h period. In the case of FnBPA-positive lactococci, there was an increase in pathogen concentration both in vegetations and in spleens. The imaging observations indicated that while ClfA-positive lactococci were restricted to the vegetations, FnBPA-positive lactococci also infiltrated the surrounding tissue, thereby elucidating the capacity of FnBPA to induce cell endocytosis in vitro. FnBPA exhibits the capacity to bind to fibrinogen and fibronectin-binding regions [95]. Therefore, to ascertain the role of these two selective functionalities in causing infection, the experiment involved two main stages. Firstly, the fibrinogen binding domain was depleted from FnBPA. Secondly, the fibrinogen binding domain of ClfA was integrated with that of FnBPA. These steps were taken to ascertain the effect of these integrations, both in cis and in trans. It was demonstrated that the inactivation of the fibrinogen binding domain of FnBPA did not result in alterations to fibronectin binding and cellular internalisation in vitro. However, it was found to be entirely responsible for the eradication of valve infectivity in vivo [95].

The capacity to elicit a state of infection was reinstated in cis with the integration of the fibrinogen binding domain of ClfA into truncated FnBPA, while in trans was obtained by co-expressing full-length ClfA and truncated FnBPA, utilising two discrete plasmids. Consequently, it is plausible to hypothesise that in *S. aureus* infection, the interaction of fibrinogen and fibronectin could collaborate for valve colonisation and endothelial penetration in a living organism [95,100]. In this context, the subject of particular interest and research is *Staphylococcus aureus* lineage or clonal complex 398 (CC398). This is due to the increasing identification of CC398 isolates as causative agents of severe infections, even in patients with no history of contact with animals. This has prompted further investigation, as well as the dissemination of CC398 isolates within communities and healthcare settings such as hospitals, which has raised concerns. This is further compounded by the documented evolution of CC398 strains towards increased virulence and antibiotic resistance, emphasising the imperative for sustained surveillance and monitoring of these bacterial strains. Consequently, the elucidation of the origin and emergence of this clonal complex is anticipated to significantly benefit future large-scale studies designed to detect sources of contamination and infection [101].

The presence of the bacterium *Staphylococcus aureus* in the blood (i.e., bacteremia) can result in serious complications, such as IE and osteomyelitis. In addition, it can facilitate the bacterium’s escape from the bloodstream to cause abscesses in various organs. The process by which the bacterium engages with endothelial cells plays a significant role in the development of these complications. In the present phase of the infectious process, the role of several bacterial proteins has been demonstrated. Among these, the extracellular adhesion protein (Eap) of *S. aureus* has been shown to be of particular relevance. This protein performs a variety of physiological processes, among which is the capacity to interact with a range of host glycoproteins [102,103,104,105,106].

It has been demonstrated that Eap exerts a dual role in inflammation, exhibiting both pro- and anti-inflammatory activities. However, rigorous in vivo testing of its role has been hindered by the challenges in defining its activity in mutant strains. There is compelling data indicating the pro-inflammatory function of Eap and the capacity of purified native adhesion protein of *S. aureus* to induce TNFα release in human peripheral blood, exhibiting a dose-dependent response. TNFα production has been shown to enhance *S. aureus* adhesion to endothelial cells, with a fourfold increase in adhesion observed. This process involves the interaction between protein A on the bacterial surface and gC1qR/p33 on the surface of endothelial cells. The findings of this study provide a compelling rationale for the critical role of Eap in the severity of *S. aureus* bacteremia. The genetic engineering of the strains was conducted with the objective of creating an isogenic set of strains. The process involved the inactivation and integration of the Eap gene, with the insertion of an intact copy of the gene being undertaken elsewhere on the bacterial chromosome. The utilisation of a mouse bacterial infection model provided evidence that Eap-expressing strains result in more severe infections. This finding suggests that Eap plays a significant role in invasive disease [103,105,106,107].

The process of bacterial colonisation has been shown to be a significant factor in the initiation of additional cycles of endothelial damage and thrombus deposition, which ultimately result in the implantation of infected vegetation [108,109]. During this stage, the development of a biofilm, which is formed by a multilayer comprising a bacterial aggregate containing a polysaccharide linked with a protein matrix, assists in the persistence of the bacteria and leads to an increase in antibiotic resistance [108,109].

- Surface molecules of *Staphylococcus aureus*

Recent experimental models have demonstrated the significant role of the surface molecules of *Staphylococcus aureus* in the adhesion process to vascular endothelium. This process has been identified as a primary component in the pathogenesis of IE. Furthermore, intensive investigation has focused on the ability of these molecules to also launch endothelial procoagulant and pro-inflammatory responses, which lead to the development of IE. The molecular mechanisms that underpin the ability of these molecules to elicit endothelial procoagulant and pro-inflammatory responses, which are pivotal in the development of IE, have been the focus of extensive research [95,110,111,112,113].

A study examined the distinct capabilities of three significant surface proteins present on the superficial layer of *Staphylococcus aureus*. Fibronectin-binding protein A (FnBPA) and B (FnBPA), in conjunction with clumping factor A (ClfA), facilitate the bacterial adhesion mechanism that differentiates cultured human endothelial cells (ECs) when interacting with *Staphylococcus aureus* [110]. In a similar manner, these molecules have been demonstrated to promote phenotypic and functional changes in ECs. The method employed involved the use of a non-invasive surrogate bacterium, *Lactococcus lactis*, which, through the process of gene transfer, expressed staphylococcal FnBPA, FnBPA, or ClfA molecules. The presence of FnBPA- or FnBPB-positive recombinant lactococci has been shown to result in an increase in the infection of ECs, reaching a magnitude that is 50- to 100-fold higher than that observed in the absence of these molecules. The investigation yielded further significant findings, including the activation of endothelial cells (EC), the production of interleukin-8 (IL-8) associated with the concomitant adhesion of monocytes, and an augmentation of surface expression of intercellular adhesion molecule-1 (ICAM-1) and vascular cell adhesion molecule-1 (VCAM-1). Conversely, infections induced by ClfA-positive lactococci did not result in EC activation. The significant action of FnBPA-positive *L. lactis* resulted in a substantial tissue factor-dependent endothelial coagulation response [110]. This response was enhanced by cell-bound monocytes, suggesting a complex interplay between microbial agents and the endothelium. The evidence indicated that *S. aureus* FnBPs, but not ClfA, contributed to invasiveness and pathogenicity in non-pathogenic *L. lactis* microorganisms, suggesting that bacterium–EC interactions mediated by these adhesins were significantly associated with inflammation and procoagulant activity at infected endovascular sites [110,114].

The experimental endocarditis induced by *Staphylococcus aureus* was modelled on actual cases of the condition. It was anticipated that sequential fibrinogen binding would be crucial to valve colony formation, and that the paramount action of fibronectin-binding would lead to endothelial invasion. The ability of these processes to persist is attributed to the action of peptidoglycan-attached adhesins. The role of fibronectin-binding protein A (FnBPA) in this context has been demonstrated to be crucial in facilitating a synthesis between these two specific properties, in conjunction with the binding of elastin, thereby promoting experimental endocarditis. The role of the minimal subdomain of FnBPA in promoting cell invasion in vivo endocarditis has been a subject of much recent research [115]. In this study, FnBPA was expressed in *Lactococcus lactis* and its properties were tested both in vitro and in animals. The subdomain responsible for the manifestation of IE comprised 127 amino acids and formed the fulcrum of the FnBPA fibrinogen-binding and fibronectin-binding regions, thereby conferring the capacity to bind to these proteins [115].

Whilst experimental evidence in animals supported the substantial role of fibrinogen binding in the induction of endocarditis, the work of fibronectin binding was not significantly associated with this condition. Conversely, in terms of disease severity, both types of binding were deemed critical. Furthermore, the concomitant presence of fibrinogen binding and fibronectin binding led to a substantial augmentation in the infectious infiltration of cultured cell lines, underscoring a pivotal attribute associated with the severity of endocarditis [115]. It can therefore be concluded that the concept of a serial process involving fibrinogen binding and fibronectin binding, which promotes both colonisation and invasive growth, has provided novel and unexpected insights into the interconnected roles of fibrinogen binding and fibronectin binding in the context of endocarditis, with regard to both functional anatomy and the underlying pathogenetic processes. This refined and hitherto unanticipated attribute of FnBPA represents a significant advancement and paves the way for the formulation of anti-adhesin strategic initiatives [115,116].

- The way the endothelial cell surface works.

Researchers found that certain proteins found in bacteria can help them stick to molecules on the surface of cells. These proteins work together with other proteins found in the body’s connective tissue, such as fibrinogen, fibrin, fibronectin, and von Willebrand factor [112]. Ultra large von Willebrand factor (ULVWF) plays a key role in the early stages of a heart infection caused by *Staphylococcus aureus* in patients whose hearts are still intact. Using heparin and ADAMTS13 to reduce ULVWF production could open up new ways to treat infective endocarditis [113].

**Figure 4:**
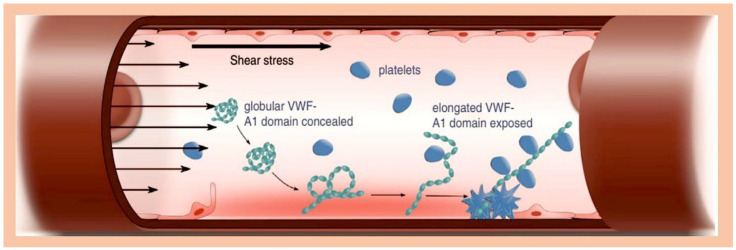
As illustrated in Figure 4, shear stress-mediated binding of platelets to von Willebrand factor (VWF) is a process of paramount significance in haemostatic pathways. VWF, found in either a secreted or circulating form in the bloodstream, is utilised by endothelial cells. However, due to the A1 domain’s crucial role in platelet binding being concealed during the globular state of VWF, platelet–VWF interactions are rendered impossible. * However, in circumstances involving endothelial damage or inflammation, VWF is retained upon the endothelium, becoming exposed to the shear stress of flowing blood. This elongation process subsequently exposes the A1 domain, allowing it to interact with the GPIb receptor on platelets, thereby inducing a reduction in platelet velocity and a partial activation response. A comparable phenomenon has been observed in the context of *Staphylococcus aureus* binding to the A1 domain of VWF, highlighting a potential universal mechanism underpinning platelet-mediated responses to injury or inflammation. *The progressive movement of black arrows from the periphery towards the centre is suggestive of laminar flow, indicating the dynamic interaction of von Willebrand Factor (vWF) and platelets within the vascular system. The presence of a solitary black arrow signifies adherence to the endothelium in the context of peripheral laminar flow. Abbreviations in the text.

Three recently published studies looked at how three different substances (ClfA, FnBPA, and von Willebrand factor (vWF)) work together to help bacteria called *Staphylococcus aureus* stick to certain cells in the body [117,118,119]. These studies show that these substances are very important in promoting IE. In a new report, scientists revealed that a protein called vWbp helps bacteria called *S. aureus* stick to blood vessels. They found that vWbp works with another protein, SrtA, to help *S. aureus* stick to a special surface of blood vessels. To test this, the scientists used different types of *S. aureus* and other proteins, as well as a type of bacteria called *Lactococcus lactis* that can produce these proteins. The researchers suggest that *S. aureus* first latches on to the vessel’s inner wall via VWF. It then secretes vWbp, which helps the bacteria stick to VWF under the influence of blood flow. Finally, vWbp interacts with VWF and a surface protein dependent on sortase A (ClfA). This anchors *S. aureus* to the vessel’s inner wall [117]. The same group looked at how blood flow and plasma affected the binding of two proteins, ClfA and FnBPA, and their sub-domains. They used a specially altered version of the bacterium *Lactococcus lactis* (*L. lactis)* to express these proteins. The results showed that the proteins stuck to the cells in the same way in both static and flowing conditions. They also noticed that when there was no plasma present, -ClfA attached to fibrinogen increased with shear forces, but attachment to fibrin did not have the expected result [118,119]. The degree to which *L. lactis*-FnBPA sticks to fibronectin and *L. lactis*-ClfA sticks to fibrinogen is similar to the degree to which *L. lactis*-ClfA sticks to coated vWF domain A1, in the presence of vWF-binding protein (vWbp) [118]. Interestingly, the ability of *L. lactis*-ClfA to stick to activated EC-vWF/vWbp in plasma dropped by 80% in just 10 min. This was linked to the breakdown of vWF by disintegrin-mediated and metalloproteinase-mediated mechanisms involving thrombospondin motif type 1, member 13, fewer *L. lactis*—FnBPA cells stuck to blood cells when plasma was absent, and more *L. lactis*—ClfA cells stuck to blood cells when plasma was present. In plasma, *S. aureus* sticks to active endothelium via two pathways: a fast but short-lived vWF/vWbp pathway and a stable fibrinogen pathway. These results showed that stopping the interaction between ClfA and fibrinogen could be a good extra treatment for infective endocarditis (Figure 4) [118,119]. 

*Staphylococcus aureus* is a bacterium that can cause problems in the blood vessels. It can lead to the death of blood vessel cells and cause the blood vessels to become damaged. We now understand that *Staphylococcus aureus* plays a key role in causing IE because it helps to spread infection through a protein called ClfA, which is found on the cell wall of *S. aureus* [119]. However, we now understand more about the role of secreted plasma coagulation factors, such as staphylo-coagulase (Coa), and the protein-binding von Willebrand factor (vWbp). In rats with aortic valvular vegetations caused by catheterisation, coagulase-positive staphylococci (Coa-positive staphylococci) and *Staphylococcus aureus* encode a von Willebrand factor-binding protein (vWbp) that has been observed to cause IE [119]. The function of vWbp in this process was the subject of a comprehensive investigation, which employed the model bacterium *Lactococcus lactis*. A number of these bacteria were genetically modified in a laboratory setting to enable the synthesis of coagulase. The investigation involved the use of various bacterial strains, including *S. aureus* Newman Δcoa, ΔvWbp, ΔclfA, or Δcoa/ΔvWbp/ΔclfA. It was observed that the presence of vWbp in *L. lactis* enhanced its capacity to infect valves, in contrast to other comparable bacteria. The expression of ClfA augmented the infectious potential of *L. lactis*, with the effect being independent of the co-expression of vWbp. It is noteworthy that the deletion of the Coa or vWbp genes in *S. aureus* did not result in a decrease in infectivity; however, the deletion of ClfA significantly mitigated valve involvement. Intriguingly, the activity of clfA remained unaltered by the triple deletion of Δcoa/ΔvWbp/ΔclfA. Evidence has indicated that Coa does not facilitate initial IE colonisation by using *L. lactis* as the pathogen in the absence of other crucial virulence factors. The contribution of vWbp to IE induced by *L. lactis* is indisputable, yet its influence appears negligible in the context of ClfA [119,120].

It has been established that *Staphylococcus aureus* is regarded as an extracellular pathogen. However, recent findings have revealed the capacity of these microorganisms to invade and be integrated by various host cells, including several types of phagocytes. This suggests the possibility of their existence within endothelial cells, epithelial cells, and osteoblasts. The intracellular location of *S. aureus* is consistent with the mechanisms that facilitate infection. The process of pathogens gaining entry into the host organism is facilitated by the binding of integrin α5β1, which is expressed on the membrane of the host cell. This integrin recognises fibronectin, thereby creating a bridge that facilitates the recognition between the pathogen and the host cell, leading to subsequent cell integration [121,122,123,124].

As demonstrated by Niemann et al., the internalisation tests and immunofluorescence microscopy revealed that *S. aureus* exhibited a reduced capacity to be engulfed by osteoblasts in comparison to epithelial cells. This finding is further corroborated by the observation that osteoblasts manifested elevated levels of both α5β1-integrin and fibronectin, along with a pronounced propensity to bind to the bacteria [125].

The infection process of cells by *S. aureus* was observed to result in the external introduction of fibronectin. This led to an increased rate of uptake in epithelial cells but not in osteoblasts. This finding is inconsistent with previously reported observations regarding the mechanism of uptake of the pathogen, in which integrin and fibronectin expression were identified as key factors in promoting bacterial uptake in host cells. Observations have revealed that the extracellular fibronectin enveloping osteoblasts and epithelial cells exhibits distinct organisational patterns. In osteoblasts, it has been observed to form a fibrillar network. Furthermore, a significant increase in the uptake of *S. aureus* by osteoblasts, resulting in the inhibition of fibril formation, a brief reduction in RNA-mediated fibronectin expression, and disruption of the fibronectin–fibril network, has been documented. Evidence indicates that the fibronectin fibril network exerts a significant impact on *S. aureus* uptake, suggesting a direct correlation between fibronectin’s supramolecular structure and its ability to regulate host cell interactions with the pathogen [125].

Niemann et al. propose the hypothesis that the primary function of fibronectin is not determined by its crude quantity, but instead is predominantly governed by the supramolecular structure of its constituent molecules. Upon deposition on the eukaryotic cell surface, these molecules assume an instrumental role in the process of bacterial uptake by host cells. The findings can provide a rationale for the significant variability observed in the efficacy of *S. aureus* absorption across different host cell types. Furthermore, the discrepancies detected in vivo between the progression of bacterial infections and the location of bacteria in various clinical settings [125] merit further investigation [126,127,128].

The molecular mechanisms that underlie the pathogenicity of *S. aureus* are linked to the expression of various virulence factors, including proteins that facilitate adherence to the host plasma and extracellular matrix proteins. Evidence indicates that IsdB-expressing bacteria bind to both soluble and immobilised vWF [129]. More recently, the iron-regulated surface determinant B (IsdB) protein, besides being involved in iron transport and vitronectin binding, has been shown to be involved in inter-protein interactions with von Willebrand Factor (vWF) [130].

The interaction between IsdB and the recombinant vWF was shown to be reversible, with its inhibition by heparin and reduction in response to high ionic strength being key observations of this study. Furthermore, the administration of ristocetin, an allosteric agent known to promote the exposure of the A1 domain of vWF, resulted in a substantial enhancement of the binding between IsdB and vWF, thereby providing a significant experimental outcome. The findings indicate that IsdB binding and *S. aureus* adhesion are significantly inhibited by a monoclonal antibody against the A1 domain, as well as IsdB-reactive IgG isolated from patients experiencing staphylococcal endocarditis. This corroborates the hypothesis that IsdB plays a pivotal role in facilitating the adhesion of *S. aureus* and its subsequent colonisation of the endothelium. Consequently, IsdB emerges as a promising therapeutic target [130].

### 3.6. Immunity to Staphylococcus aureus Infection

**Figure 5:**
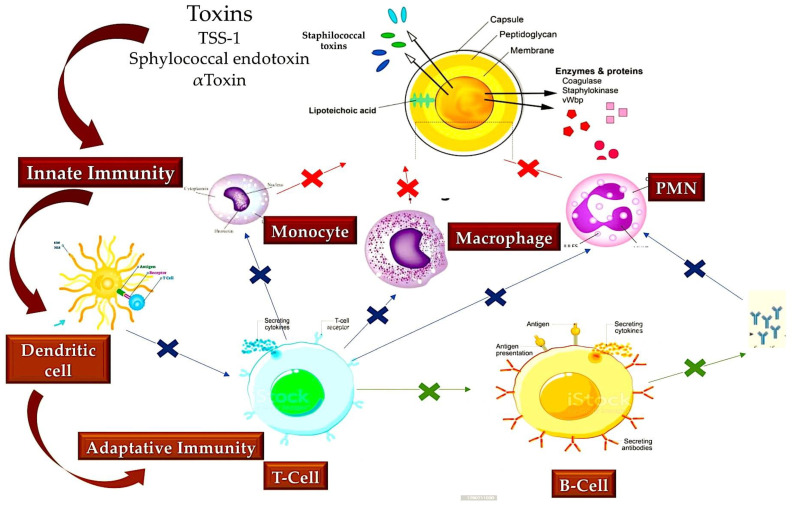
Staphylocytotoxins have an interference function (illustrated by the great blue arrow) with regard to the cells of both the innate (illustrated by the dark red box) and adaptive (illustrated by the green box) immune response. The cytoxins (TSS-1, staphylococcal endotoxin, and α-toxin) are cytolytic and can lyse immune cells (illustrated by the PMN, monocytes, and macrophages involved in the clearance of *S. aureus* [illustrated by the red arrow]). Furthermore, the ability of cytotoxins to impair the function of adaptive immune cells (green arrows), represented by both T and B lymphocytes, is well documented. Finally, it is noteworthy that cytotoxins can impair the delicate balance between innate and adaptive immune cells (blue arrows). Abbreviation: TSS-1, Toxic Shock Syndrome-1. From Nappi et al. Ref. [15].

*Staphylococcus aureus* is capable of expressing numerous virulence factors, both on its surface and in its secretory substances. Once these factors are activated, they exhibit a high degree of capacity to counteract the immune defence mechanisms of the host [131,132]. The most significant virulence factor of *S. aureus* is the accessory gene regulatory system (Agr), which functions in the detection of pathogen population density, known as quorum sensing. Despite the established knowledge that Agr functions by regulating the expression of phenol-soluble modulins (PSM) with activity against immune cells such as keratinocytes (KCs), the temporal execution of this mechanism remains to be elucidated [133]. The innate immune response has been observed to elicit a reaction from dead KCs, leading to the production of a physical defence mechanism comprising antimicrobial peptides such as human β-defensin 2 and 3, cathelicidins, and RNase 7. This defence mechanism functions by means of bacteriostatic effects to counteract infection by *S. aureus*. As has been documented, the antibacterial function of KCs is initiated by pattern recognition receptors (PRRs), including TLRs and nucleotide-binding oligomerization domain (NOD) proteins. These two surveillance frameworks are capable of identifying molecular patterns linked with invading pathogens (PAMPs), thereby facilitating the prompt initiation of defence mechanisms against *S. aureus* [134,135]. As illustrated in Figure 5, the innate immune response is further enhanced by the activities of various cell types, including dendritic cells, B and T cells, macrophages, mast cells, natural killer (NK) cells, plasma cells, and fibroblasts located within the dermis [136,137].

It has been established that *S. aureus* infection is facilitated by several underlying processes, with the initial phase involving the disruption of the innate immune system. The subsequent phases involve the entry of the pathogen into the bloodstream and its dissemination into the host tissue following its exit from the bloodstream. These processes are facilitated by the unique functions of surface-expressed virulence factors that interact with the endothelium, blood, and the extracellular matrix. Initially, the binding of FnBPA and FnBPB to fibronectin is followed by their interaction with integrin α5β1 on the surface of the vascular endothelium, thus initiating cell invasion and transmigration. Subsequently, wallethic acid (WTA) and lipoteichoic acid (LTA) of *Staphylococcus aureus*, polymers that constitute the bacterial outer membrane, interfere at this stage to promote the staphylococcal penetration of the host cells. In the second step of the process, staphylococci have been observed to induce the formation of fibrin thrombi (i.e., clots of blood) through the activation of the agglutination process mediated by Coa/vWbp and ClfA. These bacteria have also been shown to bind to von Willebrand factor (vWF) on the endothelial surfaces, thereby generating polymers such as ultra large vWF (ULVWF). The third phase in this process results in the secretion of the Hla toxin by the *S. Aureus* bacterium. This toxin binds with ADAM10, which leads to the cessation of the physiological barrier functions of the endothelium in the vascular system. The final phase involves the activation of the trojan horse model, where neutrophils that have phagocytosed *S. aureus* release bacteria into the tissues of the host.

IsdA has been shown to promote the survival of *S. aureus* on human skin. Analysis of deletion constructs indicates that the IsdA domain, which is responsible for the observed reduction in hydrophobicity and resistance to innate immune defence molecules, is distinct from the NEAT domain and located towards the protein’s C terminus [138,139]. A key finding in the latest research from Clarke et al. [140] is the realisation of the significance of sebum lipids as a dynamic component of skin innate immunity. Similarly to the effects of antimicrobial peptides, sebum lipids and fatty acids are induced in response to injury or microbial stimuli via toll-like receptor-dependent pathways [141,142]. Research on knockout mice deficient in biosynthesis of these components indicates that they are unable to restrict bacterial proliferation [142,143]. In addition, reduced levels of certain sebum fatty acids and antimicrobial peptides have been observed in atopic dermatitis (eczema), an inflammatory skin condition that is predisposed to recurrent bacterial infection (Takigawa et al., 2005, Ong et al., 2002) [144,145].

- Staphylo cytotoxins can change how the immune system works.

Given the broad targeting of immune cells by *S. Aureus* during the infection process, the release of cytotoxins by the pathogen is of critical importance. These include leukotoxins such as LukED and LukAB, gamma hemolysins such as HlgAB and HlgCB, and PVL. LukAB has been observed to be efficacious exclusively on human polymorphonuclear leukocytes (PMNs) [138] and has the capacity to destroy dendritic cells, monocytes, and macrophages. LukED has been shown to both detect and induce the death of a range of cellular receptors, including C-C chemokine receptor 5, dendritic cells, macrophages, and lymphocytes [146,147]. At the micromolar scale, however, the significance of PSM and Hla becomes evident. PSM exerts a notable capacity to elicit neutrophil death following phagocytosis [148]. Moreover, it has been observed to engage in an interaction with disintegrin A and metalloprotease 1 (ADAM1), thereby potentiating the elimination process of monocytes, macrophages, neutrophils, and T cells [149]. A key point to consider is the role of cytotoxins, which promote the spread of S. aureus, and how this is different to *S. aureus* evading the host’s immune response. Cytotoxins can weaken both the natural and acquired immune responses, enabling *S. aureus* to multiply within the host (Figure 5) [150].

- Imbalance in how B cells and T cells work together.

The evasion of host immune surveillance by *S. aureus* is facilitated by SpA proteins, which are integrated into the architecture of the *S. aureus* wall and released during the growth of the pathogen. Silverman et al. and Goodyer et al. [151] have provided evidence for the involvement of five domains in the SpA that are implicated in the binding of immunoglobulins. These domains have been shown to bind to the IgG Fcγ and Fab domains of the VH3 IgG and IgM clan, thereby facilitating the polyclonal proliferation of B cells and the consequent activation of the superantigen SpA. This process is driven by the cross-links of the B cell receptors, which have been identified as a critical mediator of SpA-induced immune evasion.

A study of the stages of the infection revealed a range of growth responses, which in turn evoked a variable expression of SpA. This in turn led to the release of Hla toxin, which in turn triggered specific B lymphocytes in areas distant from *S. aureus*. The following immunological rationale underpins the human propensity to produce antibodies against Hla, notwithstanding the prevalence of SpA strains. It is imperative to acknowledge the role of the cell wall of the pathogen in the mediation of Hla release. It has thus been demonstrated that the superantigen activity displayed by SpA proteins can exert an influence in a region remote from the site of infection, thus constituting a pivotal element in the context of vaccine formulation. A particular effect that has been documented pertains to SpA proteins that evade the recognition of B cells, culminating in a state designated as “lethargy”, which constitutes a standard initial response to the antigen. In such instances, the B cells may fail to elicit a secondary signal, thereby hindering their activation and consequently inducing a state of shock known as “anergy”. The process of anergy has been observed in two distinct areas of research. Firstly, it has been identified in the context of *Staphylococcus aureus* colonisation. Secondly, it has been observed to occur during the persistence of infection. Thirdly, the phenomenon has been linked to the weakening of T cell help. This weakness is attributed to the impact of superantigens on T cell and cytotoxins, which has been shown to reduce their affinity for antibodies [152,153,154].

### 3.7. Infective Endocarditis, Platelet, and Antiaggregation

The majority of pathogens that lead to IE bind to and activate platelets, subsequently utilising these cells as a conduit to overcome shear stress. *Staphylococcus aureus*, for instance, has been demonstrated to possess a plethora of mechanisms by which to modulate platelets. Furthermore, *Staphylococcus aureus* has been observed to secrete a fibrinogen-binding adhesin, designated Clumping factor A (ClfA), which facilitates the binding of bacteria to platelets. This process results in the induction of αIIbβ3 clustering and subsequent platelet activation [81,94]. As previously outlined in the report, cardiac damage results in the unveiling of the subendothelial matrix and the accumulation of von Willebrand factor (VWF) and fibrin, thereby enabling *Staphylococcus aureus* to bind directly to the valve leaflets via the adhesins ClfA and vWbp. The role of platelets in this process is minimal. In the case of cardiac valve inflammation, endothelial cell activation is known to trigger widespread von Willebrand factor (VWF) release. This, in turn, prompts platelet binding to the valve. It is hypothesised that platelets may capture bacteria either before or after adhesion to the inflammatory valve [94].

The use of antibiotic prophylaxis is currently advised for patients with high-risk IE, but this infection remains challenging to treat and is statistically associated with a higher mortality rate. Additionally, there are concerns about the administration of antibiotics due to their low efficacy, which contributes to the increasing rate of infection and the selection of antibiotic-resistant strains. The utilisation of pharmaceuticals that target platelets has emerged as a promising therapeutic modality [155,156,157,158,159,160].

In light of this scenario, the requirement for novel pharmacological interventions persists as a salient challenge in the management of IE. The therapeutic interventions encompassed the administration of aspirin [157,158,159] antagonist of the platelet receptor P2Y12 [157,158,159,160] and Tifacogin (recombinant tissue factor pathway inhibitor) [161] in patients experiencing severe sepsis, with the objective of evaluating their efficacy and safety.

Platelets play a pivotal role in the primary stage of infective endocarditis, functioning as primary immune effectors. Studies conducted in a laboratory setting have demonstrated the pivotal role of platelets in the early stages of infective endocarditis, marking the initial phase of the immune system’s response. This initial response is characterised by the engagement of pathogens with platelets, emphasising the importance of neutralising platelet antimicrobial activity as a key therapeutic objective [155,156]. In experimental in vitro and animal models, the use of aspirin, either as a monotherapy or in combination with ticlopidine, has been demonstrated to reduce bacterial–platelet interactions, thereby preventing the development of vegetation. The therapeutic effects were observed to be selective, affecting Gram-positive bacteria in a manner that differed from the effects on *Staphylococcus aureus*, and including *Enterococcus faecalis* and *Streptococcus gallolyticus* [162,163].

These findings are encouraging; however, further research is required to clarify the role of aspirin in the treatment of patients with infective endocarditis. Clinical studies on the outcomes of aspirin-based medical therapy in this patient population remain inconclusive and require further examination. Indeed, the utilisation of aspirin as an adjuvant treatment for endocarditis is one of the few interventions that have been the subject of study in a randomised clinical trial [164]. In this RCT, 115 patients with the condition of endocarditis were divided into two groups. The first group was administered a placebo, while the second was administered a high dose of aspirin (325 mg/day for a period of four weeks). The study found that treatment with aspirin had no effect on clinical outcomes and was associated with a trend towards a higher incidence of bleeding. Therefore, both the European and American guidelines recommend that antiplatelet agents should not be used in the therapeutic management of infective endocarditis [165,166].

The findings have led to a degree of uncertainty regarding the benefits of antiplatelet agents in the prevention of IEs. In addition to aspirin, ticagrelor, an antagonist of the platelet receptor P2Y12, has been shown to have a therapeutic effect. The synergy arising from the combination of the potent, well-established antiplatelet activity with the robust antibacterial properties is of particular significance in the context of Gram-positive bacteria, including methicillin-resistant *Staphylococcus aureus* [160]. It has been hypothesised that ticagrelor, a drug that exhibits strong binding affinity to platelets, may facilitate more efficient permeation into the core of an endocarditis lesion in comparison to other antibiotics. Furthermore, the antiplatelet properties of ticagrelor could potentially impede the recruitment of additional platelets and bacteria into the expanding vegetation. However, it should be noted that these initial observations require further investigation and validation.

The findings of both the experimental study on mice and the clinical investigation failed to demonstrate the efficacy of aspirin and ticagrelor in eradicating *Staphylococcus aureus* bacteremia. The primary concern pertains to the necessity for a substantial number of individuals to possess sufficient statistical power, unless a patient group with a very high risk can be identified. Additionally, given the low incidence of endocarditis, the number of individuals requiring treatment would likely exceed the number of individuals harmed by any intervention.

## 4. Perspective

The advent of antibiotics has led to a favourable prognosis for patients afflicted with infective endocarditis, a condition that previously resulted in a high mortality rate. In the contemporary era, the judicious administration of antibiotics in conjunction with prompt surgical intervention, when deemed essential, has become a critical component of effective patient management, often resulting in the preservation of life. Notwithstanding the recently published European [4] and Duke-International Society for Cardiovascular Infectious Diseases Criteria for Infective Endocarditis [5] of a commendable scientific standard, the mortality rate of endocarditis in general (20–30%) and of *Staphylococcus aureus* endocarditis in particular (30–40%) continues to be a matter of concern [6]. It is of particular interest that, despite the advances in medical technology, there has been no improvement in the clinical outcome of endocarditis over the course of the preceding decades [81]. This absence of advancement highlights the necessity for novel, innovative therapeutic interventions. The coagulation system has been identified as a promising therapeutic target due to its critical role in the development of endocarditis. Furthermore, there is a plethora of highly effective pharmaceutical agents available that target coagulation, thus rendering it an attractive therapeutic avenue.

Indeed, as evidenced by numerous studies, the utilisation of fibrinolytic drugs, anticoagulants, and antiplatelet agents in the management of infective endocarditis has been investigated [167]. Regrettably, the rarity of the condition and its diverse manifestations pose significant challenges in the execution of high-quality clinical trials in patients with IE. The quality of the evidence is frequently inadequate, consisting primarily of animal studies or retrospective cohort trials. A further issue is that, due to the elevated incidence of cerebral embolism, as many as 60% of patients with endocarditis exhibit intracranial haemorrhagic lesions, including microbleeds, when systematically assessed with magnetic resonance imaging [168]. Consequently, these patients face an elevated risk of experiencing intracranial haemorrhaging. Antithrombotic therapy is thus typically a challenging decision that necessitates careful deliberation due to its potential adverse consequences frequently outweighing its intended benefits.

## 5. Conclusions

Infective endocarditis caused by *Staphylococcus aureus* arises from a multifaceted interplay between virulence factors produced by the bacterium, processes related to coagulation, components of the innate immune system, and haemodynamics. At nearly every stage of the disease process, the coagulation system plays an instrumental role. However, its impact on the host can vary, ranging from a protective effect to a detrimental one, depending on the specific circumstances of the infection. It is evident that the therapeutic manipulation of this intricate mechanism carries inherent risks, as any disruption to the equilibrium of hemostasis may precipitate the exacerbation of the infectious condition or precipitate potentially lethal bleeding events. The aforementioned intricacies elucidate the reasons as to why it is frequently arduous to translate the findings derived from highly standardised inbred animal models to complex clinical cases observed in everyday practice. The majority of endeavours to manipulate coagulation in instances of serious microbial infections to date have proved ineffective.

The purpose of this study was to illustrate the following point by means of the presentation of the following examples. Firstly, the use of either recombinant activated protein C [169] or tissue factor pathway inhibitor [161] was examined for sepsis patients, and secondly, the use of aspirin was investigated in patients with endocarditis [164]. The outcomes of these investigations revealed no improvement in patient outcomes whilst concomitant bleeding complications were identified. Nevertheless, it can be concluded that this does not imply an absence of value in conducting further research in this area.

Further investigation into the fundamental and translational aspects of the intricate connection between infection and coagulation is imperative to attain a more profound comprehension of this multifaceted dynamic. This enhanced knowledge will facilitate the development of more precise and efficacious instruments to combat infections in their totality, and specifically, infective endocarditis.

## Data Availability

No new data were created or analyzed in this study.
